# Supplementary figures and images for: The m6A reader YTHDF2 is a negative regulator for dendrite development and maintenance of retinal ganglion cells
Source: eLife. 2022 Feb 18;11:e75827. doi: 10.7554/eLife.75827 (PMC8906807; doi:10.7554/eLife.75827)

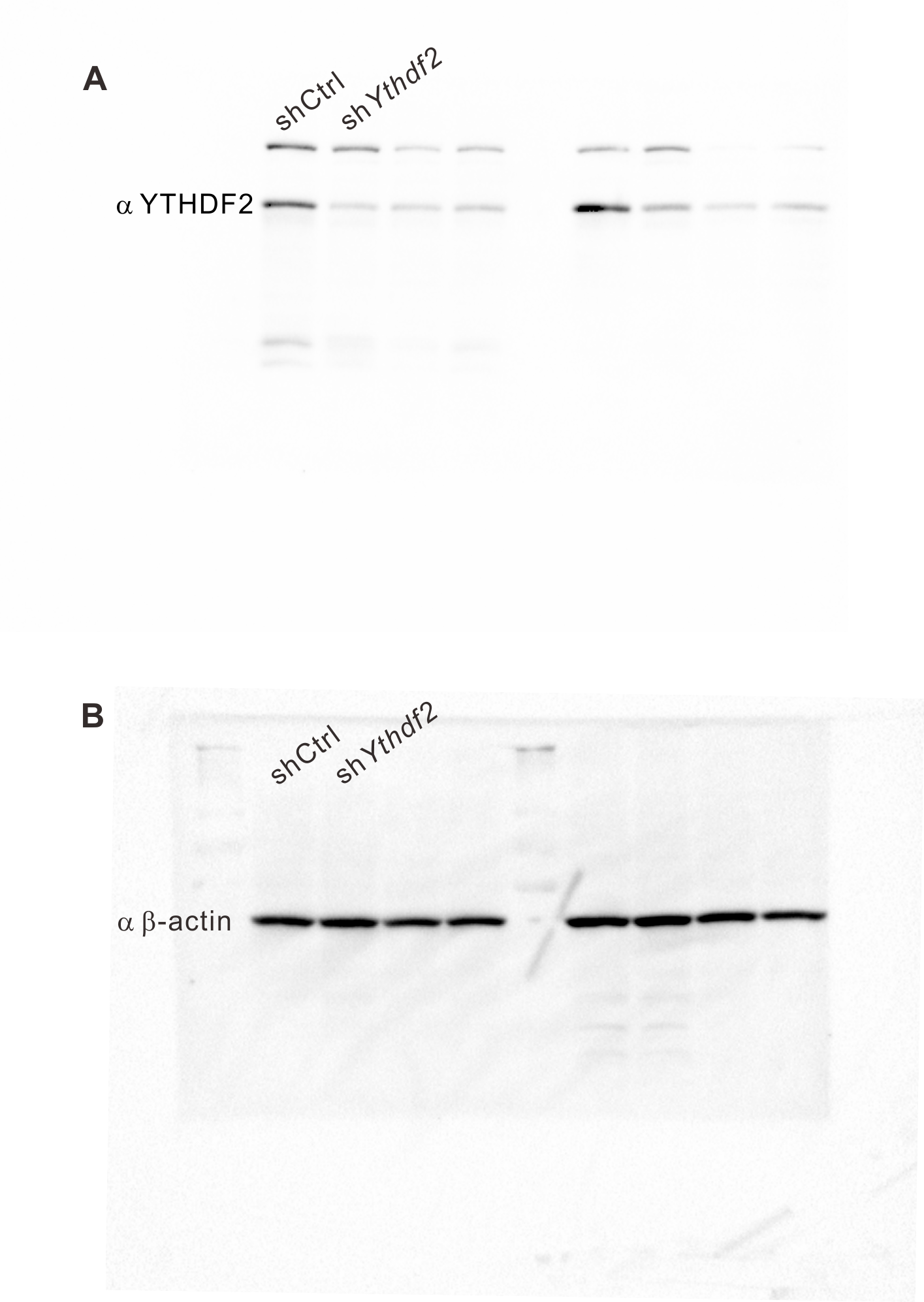

Supplement: Figure 1—source data 1. — (A) Western blotting (WB) of anti-YTHDF2 after knockdown (KD) of YTHDF2. (B) WB of anti-β-actin after KD of YTHDF2. [file elife-75827-fig1-data1.zip › Figure 1-source data 1.tif]

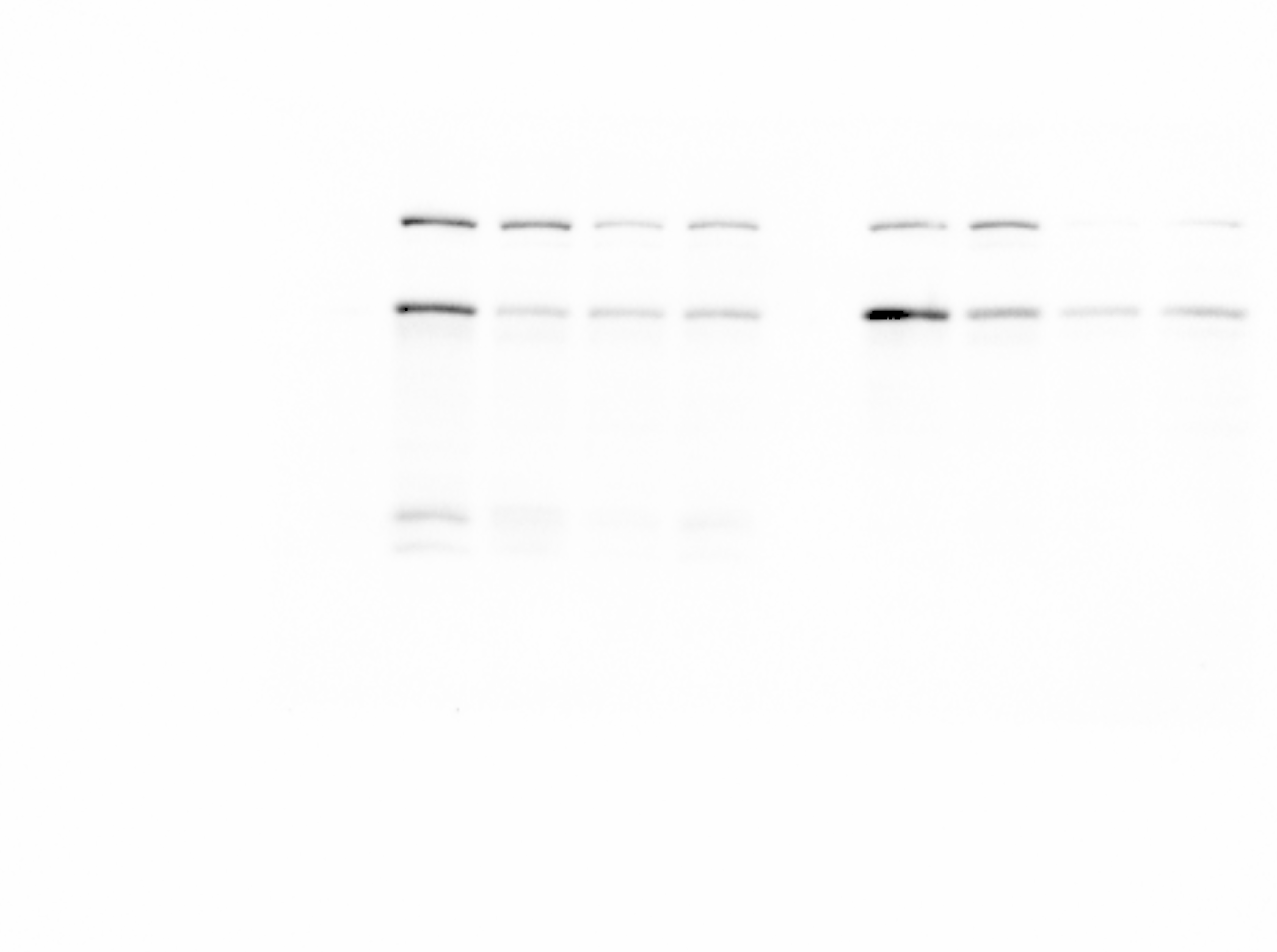

Supplement: Figure 1—source data 2. — Original file of the full raw unedited blot of anti-YTHDF2 after knockdown (KD) of YTHDF2. [file elife-75827-fig1-data2.zip › Figure 1-source data 2.tif]

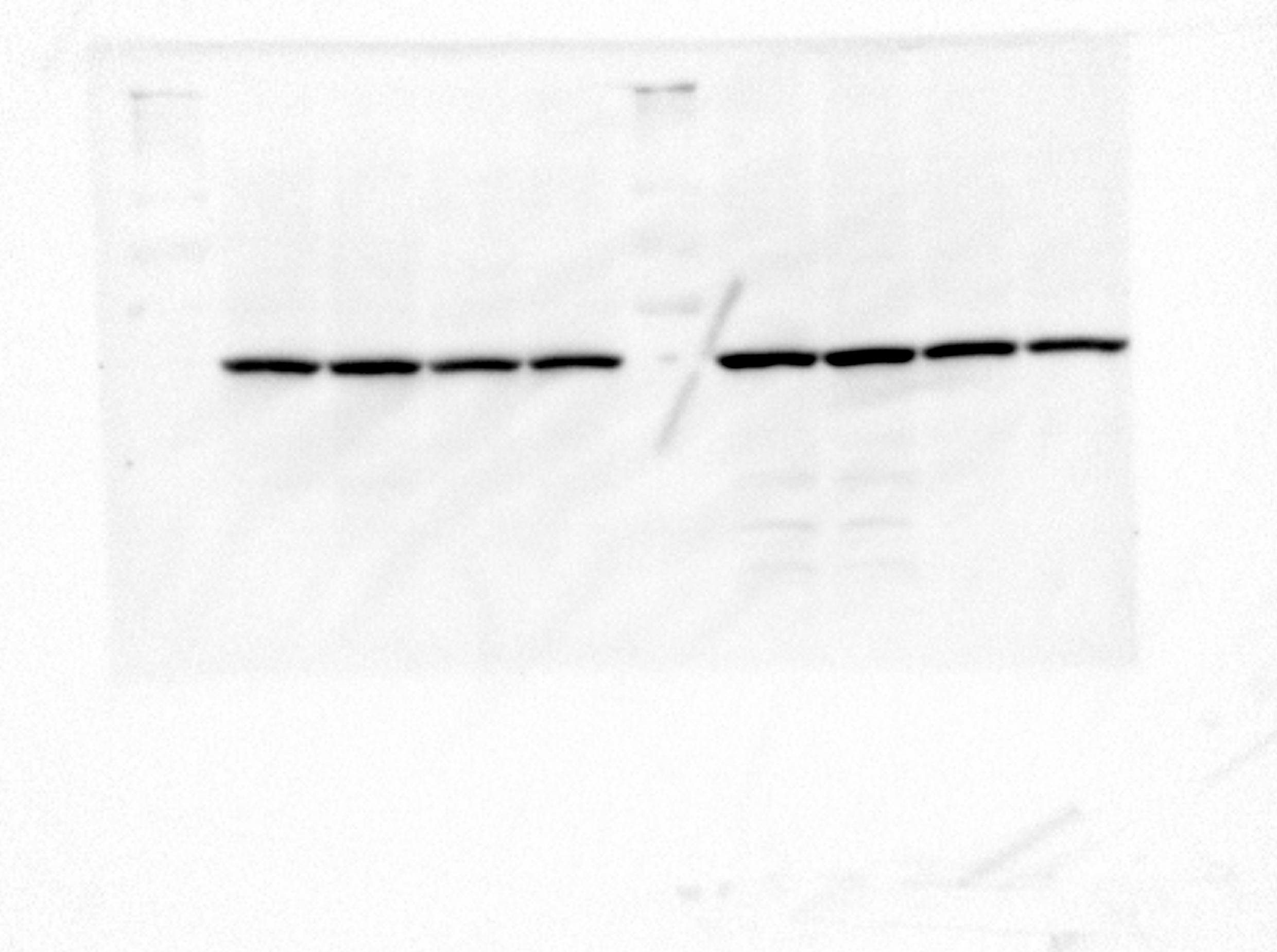

Supplement: Figure 1—source data 3. — Original file of the full raw unedited blot of anti-β-actin after knockdown (KD) of YTHDF2. [file elife-75827-fig1-data3.zip › Figure 1-source data 3.tif]

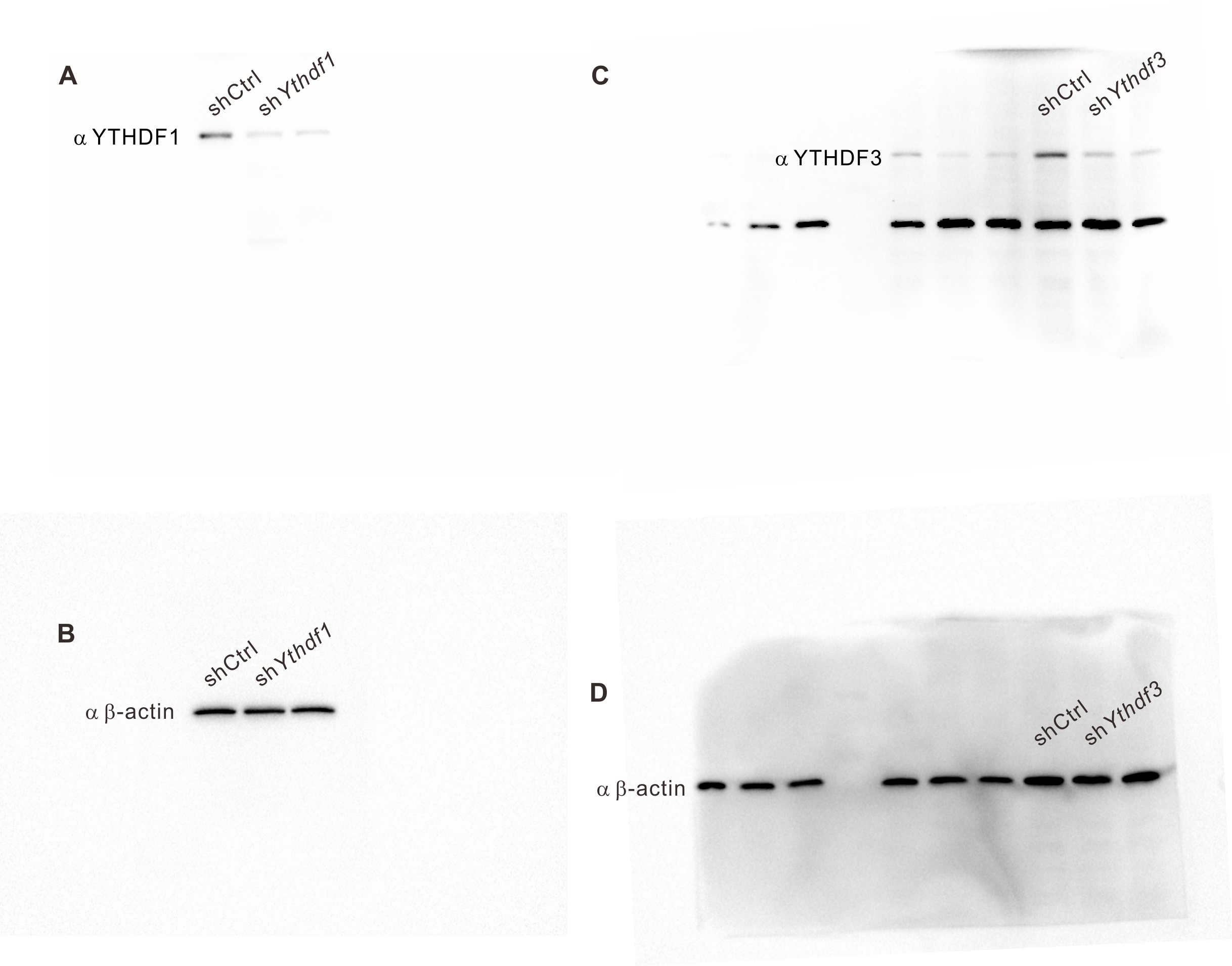

Supplement: Figure 1—figure supplement 1—source data 1. — (A) Western blotting (WB) of anti-YTHDF1 after knockdown (KD) of YTHDF1. (B) WB of anti-β-actin after KD of YTHDF1. (C) WB of anti YTHDF3 after KD of YTHDF3. (D) WB of anti-β-actin after KD of YTHDF3. [file elife-75827-fig1-figsupp1-data1.zip › Figure 1-figure supplement 1-source data 1.tif]

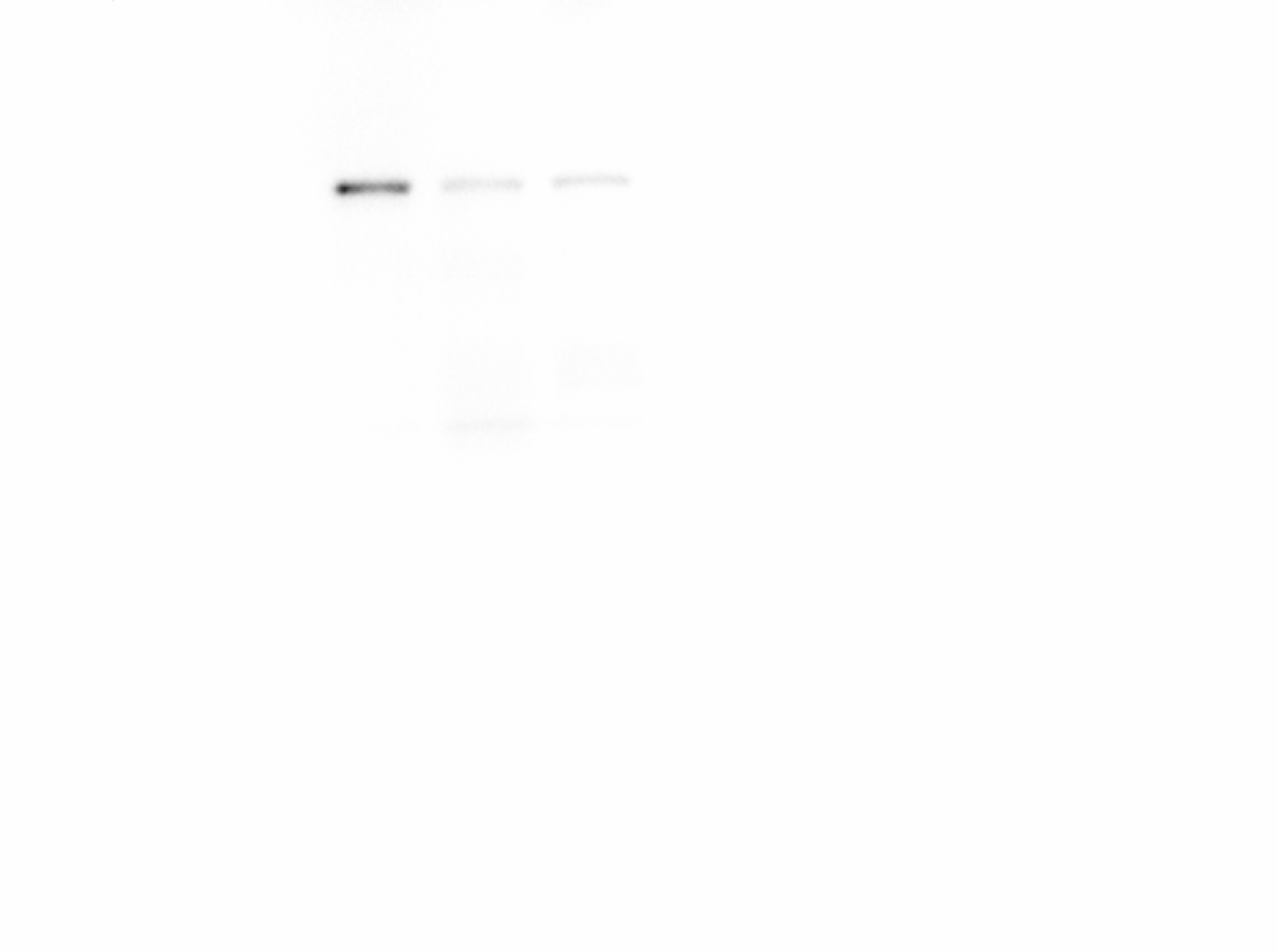

Supplement: Figure 1—figure supplement 1—source data 2. — Original file of the full raw unedited blot of anti-YTHDF1 after knockdown (KD) of YTHDF1. [file elife-75827-fig1-figsupp1-data2.zip › Figure 1-figure supplement 1-source data 2.tif]

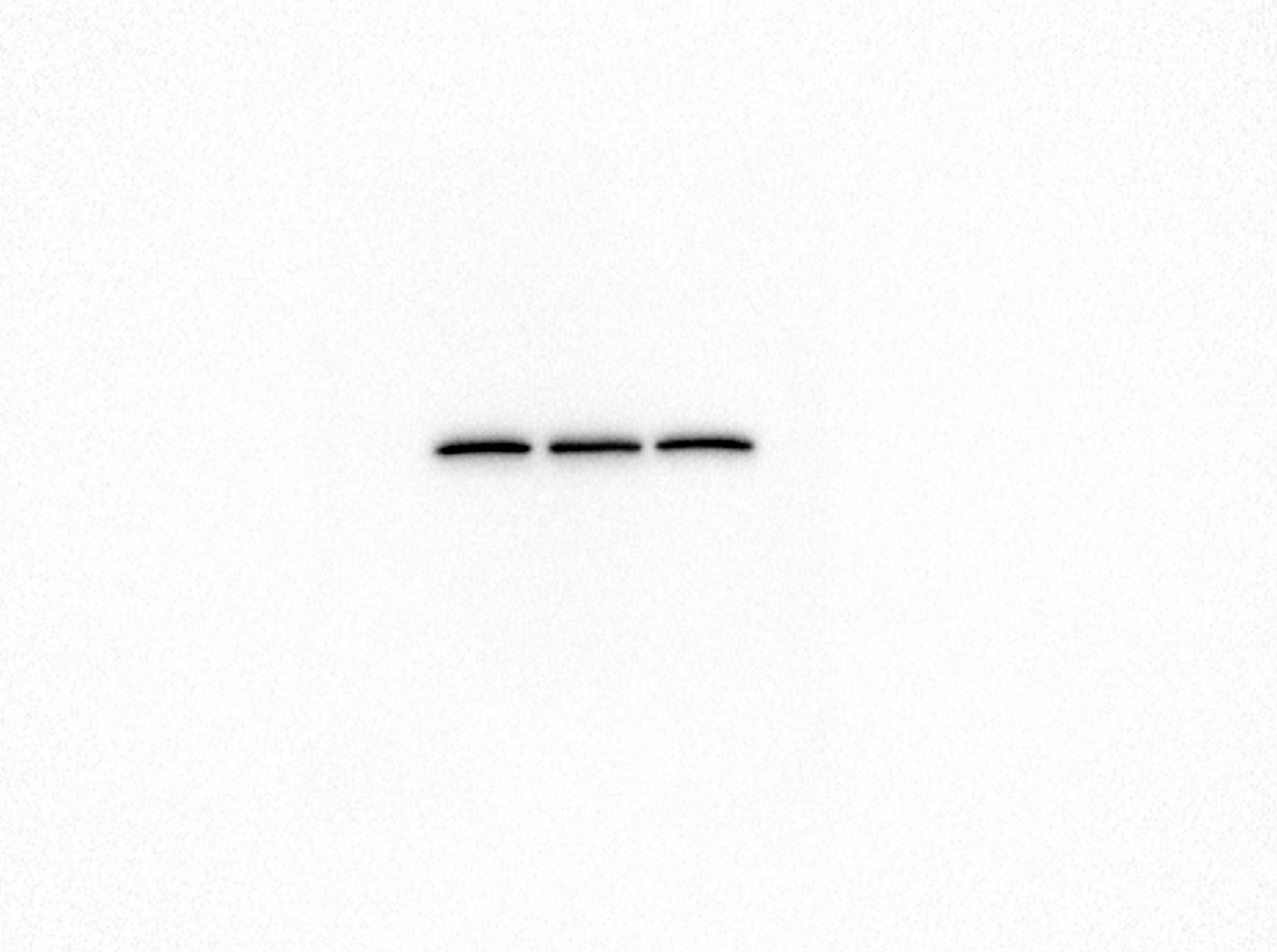

Supplement: Figure 1—figure supplement 1—source data 3. — Original file of the full raw unedited blot of anti-β-actin after knockdown (KD) of YTHDF1. [file elife-75827-fig1-figsupp1-data3.zip › Figure 1-figure supplement 1-source data 3.tif]

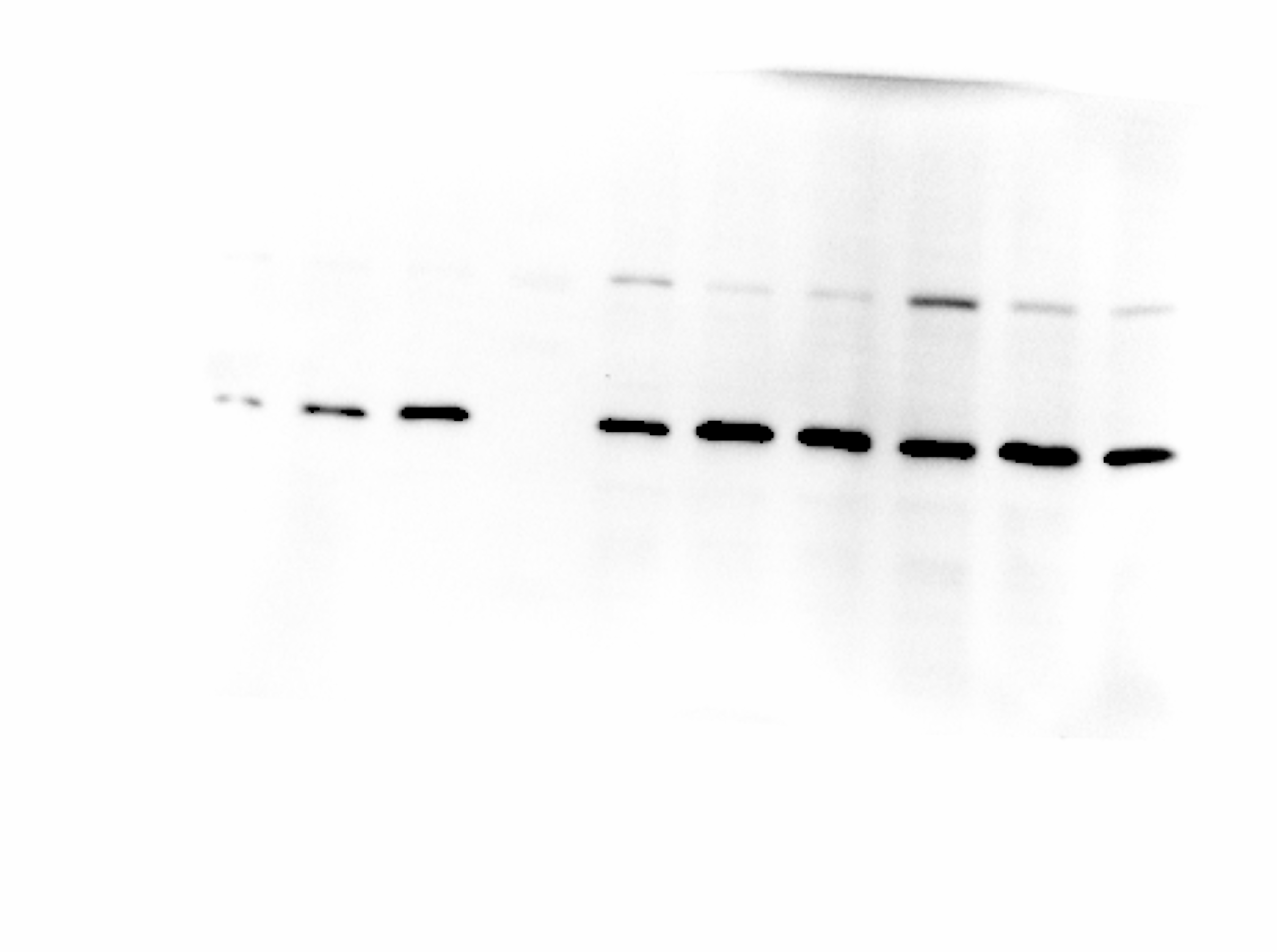

Supplement: Figure 1—figure supplement 1—source data 4. — Original file of the full raw unedited blot of anti-YTHDF3 after knockdown (KD) of YTHDF3. [file elife-75827-fig1-figsupp1-data4.zip › Figure 1-figure supplement 1-source data 4.tif]

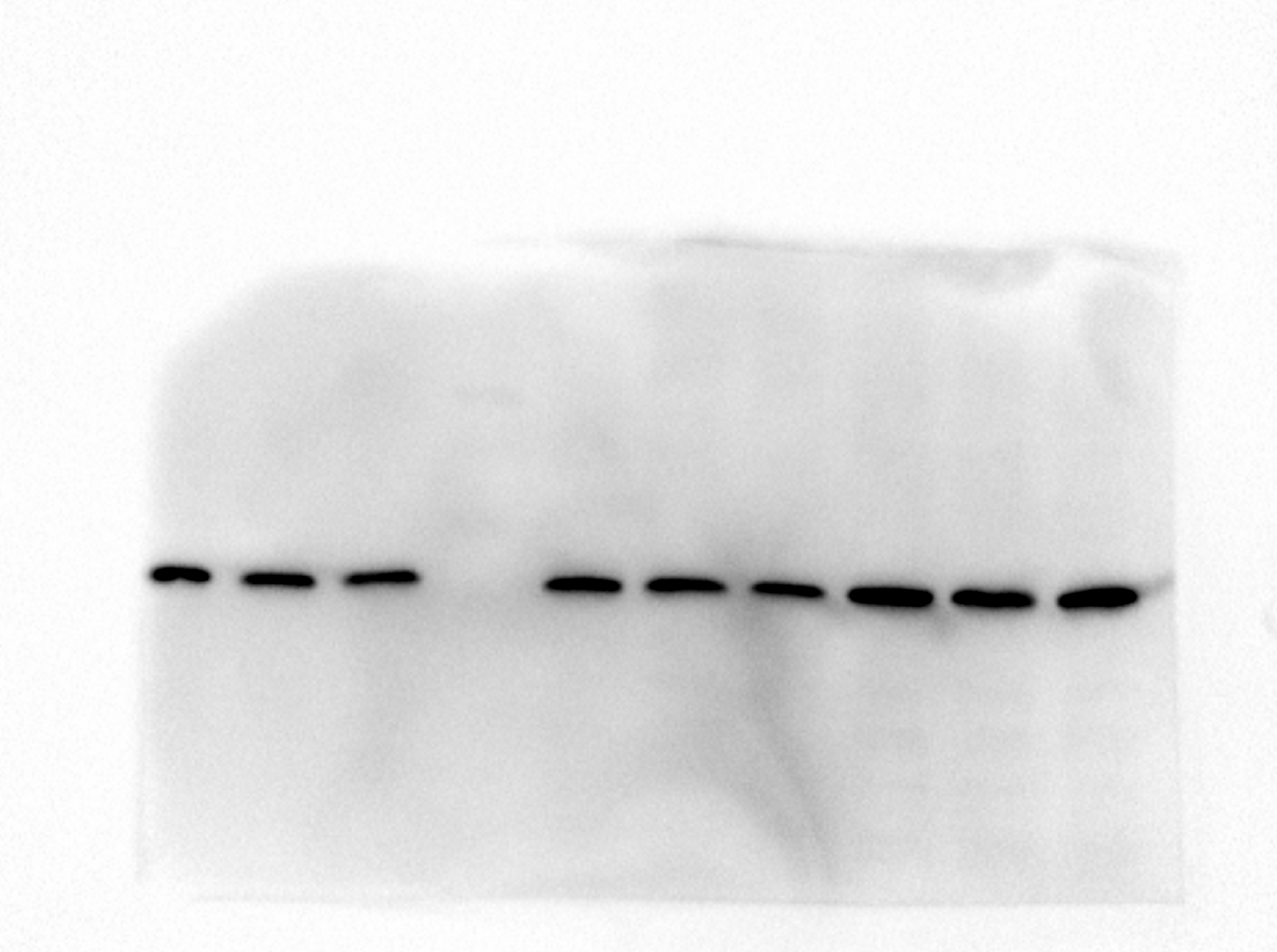

Supplement: Figure 1—figure supplement 1—source data 5. — Original file of the full raw unedited blot of anti-β-actin after knockdown (KD) of YTHDF3. [file elife-75827-fig1-figsupp1-data5.zip › Figure 1-figure supplement 1-source data 5.tif]
